# Metagenomics research on the gut microbiota of the *Marmota himalayana* of the Sanjiangyuan National Nature Reserve in Qinghai Province, China

**DOI:** 10.1016/j.bsheal.2025.09.003

**Published:** 2025-09-08

**Authors:** Ying Ma, Ziyan Li, Pengbo Liu, Youwen Wei, Ke Jiang, Yujuan Yue, Aiping Zhang, Wenlong Wang, Lingwen Li, Penghui Zhang, Xingyue Gu, Qiyong Liu, Liang Lu

**Affiliations:** a Qinghai Institute for Endemic Disease Prevention and Control, Xining 810021, China; b Bioscience and Biomedical Engineering Thrust, The Hong Kong University of Science and Technology (Guangzhou), Guangzhou 511453, China; c National Key Laboratory of Intelligent Tracking and Forecasting for Infectious Diseases, National Institute for Communicable Disease Control and Prevention, Chinese Center for Disease Control and Prevention, Beijing 102206, China

**Keywords:** Metagenomics, *Marmota himalayana*, Gut microbiota, The Sanjiangyuan National Nature Reserve

## Abstract

With the improvement of transportation and the rise of tourism on the Qinghai-Xizang Plateau, the scope of human activities has continuously expanded, increasing opportunities for contact with wildlife, also exacerbating the outbreak rate of zoonotic emerging infectious diseases. Currently, research on the gut microbiota of wildlife, especially *Marmota himalayana* (*M. himalayana*), which are reservoir hosts for plague, is scarce. In this study, we investigated the composition, function, and regional variations of the gut microbiota in *M. himalayana* based on the metagenomic sequencing of 45 fecal samples from the Sanjiangyuan National Nature Reserve in Qinghai Province. The results indicated that at the phylum level, the dominant bacterial phyla in the gut microbiota of the *M. himalayana* were *Firmicutes*, *Bacteroidota*, and *Proteobacteria*, collectively accounting for 74.16 % of the community. At the genus level, the top three most abundant genera were *Alistipes* (11.86 % ± 1.56 %), *Bacteroides* (6.68 % ± 0.95 %), and *Clostridium* (4.92 % ± 1.04 %). Kyoto encyclopedia of genes and genomes (KEGG) database annotation results showed that the most enriched functional categories of the marmot gut microbiota were metabolism, genetic information processing (GIP), and environmental information processing (EIP). These active functions played a crucial role in food digestion, nutrient absorption, metabolic balance maintenance, and pathogen defense, aiding the marmot in better adapting to the extreme environment of the Qinghai-Xizang Plateau. The study provided critical insights into host-microbe interactions, highlighting the role of microbiota in the survival and conservation of endangered species in unique habitats.

## Highlights

**Scientific questions** Increasing opportunities for contact with wildlife may exacerbating the outbreak rate of zoonotic emerging infectious diseases. This study analyzed the gut microbiota of *Marmota himalayana* with respect to nutrient transformation and immune regulation, particularly in the context of adaptation to the unique geographical environment of the intestine on the Qinghai-Xizang Plateau.
**Evidence before this study** *Marmota himalayana* had been the focus of most existing research on infectious diseases, particularly concerning the transmission of *Yersinia pestis* as an endemic herbivore of the Qinghai-Xizang Plateau. However, limited studies have reported on its gut microbiota in relation to nutritional metabolism and the regulation of immune homeostasis.
**New findings** The gut microbiota of *Marmota himalayana* is characterized by a functionally diverse composition, with *Alistipes, Bacteroides*, and *Clostridium* serving as the core dominant genera. Key antibiotic resistance genes detected include *MCR-1.2*, *SHV-100*, *MexH*, and *vanC*, all of which are associated with bacterial antibiotic resistance. Functional enrichment analysis revealed that metabolism and genetic information processing are the most prominent functional categories. These compositional features and functional attributes play critical roles in food digestion, nutrient absorption, metabolic homeostasis maintenance, and pathogen defense, enabling the marmot to better adapt to the extreme environment of the Qinghai-Xizang Plateau.
**Significance of the study** Our study reveals that a structured and metabolically active microbial community supports the physiological adaptation of *Marmota himalayana* to the extreme conditions of the Qinghai-Xizang Plateau. Additionally, it provides critical insights into host-microbe interactions, highlighting the role of microbiota in the survival and conservation of endangered species in unique habitats and its implications for biodiversity conservation.

## 1 Introduction

The gut microbiota is defined as the community of microorganisms that reside within the intestines of humans and other animals, primarily comprising bacteria, viruses, fungi, archaea, and other microorganisms. In recent years, it has emerged as one of the most captivating research focuses in the fields of microbiology, medicine, genetics, and other related disciplines. It is crucial in host nutrition, metabolism, and immunity. It synthesizes essential proteins and vitamins that the host can readily absorb while degrading and fermenting carbohydrates to generate energy for the host [1]. A healthy gut microbiota interacts with intestinal epithelial cells, forming a protective barrier that prevents pathogenic bacterial invasion and helps maintain microbiome stability [2]. Therefore, maintaining the balance of the gut microbiota is considered crucial for promoting health and preventing diseases.

The gut microbiota of wild animals living on plateaus exhibit high species diversity. The species composition, abundance, and function of the gut microbiota among plateau species are likely influenced by factors like the ecological environment, dietary preferences, and evolutionary history. While research on the global gut microbiota of herbivores has advanced significantly, studies focusing on plateau species remain scarce, especially in comparative analyses of species diversity, geographical distribution, and ecological adaptability. Current research mainly focuses on specific species such as the Tibetan antelope [3], rock sheep [4], Tibetan wild donkey [5], Tibetan sheep [6], plateau pika [7], and myospalax [8], with relatively little attention given to the gut microbiota of other plateau species. To date, limited studies have been reported on its gut microbiota community concerning nutrient transformation, immune regulation in the context of adaptation to the unique geographical environment of the intestine of the *Marmota himalayana* (*M. himalayana*), an herbivorous animal endemic to the Qinghai-Xizang Plateau.

Plague is recognized as a highly infectious, rapidly spreading, and often fatal epidemic disease caused by *Yersinia pestis* (*Y. pestis*). The Qinghai-Xizang Plateau hosts the largest and highest-altitude natural plague focus globally, with *M. himalayana* identified as the primary reservoir host [9]. *M. himalayana* serves as the primary reservoir responsible for the spread of plague among animals in the region. From 1954 to 2012, *Y. pestis* strains isolated from *M. himalayana* in Qinghai Province comprised 36.51 % (597/940) of all detected strains [10]. According to different region (DFR) profiles, the strains are primarily classified under genomovar 5 and 8 in the Qinghai Plateau [11]. Recent research indicates that *M. himalayana* populations are divided into two primary clusters, located in the southern and northern regions. Moreover, increased genetic diversity among plague hosts is positively correlated with human plague outbreaks on the Qinghai-Xizang Plateau [12].

The Sanjiangyuan National Reserve is the source of the Yangtze River, the Yellow River, and the Lancang River. The reserve is rich in unique germplasm resources and indigenous species, making it significant for biodiversity conservation and germplasm resources preservation. The Sanjiangyuan region is the primary source of human plague in China, with the high virulence of *Y. pestis*. In addition, it encompasses the most diverse isolates and is believed to be the origin of *Y. pestis* [13]. The region’s unique geographical landscape and resources highlight the scientific importance of studying gut microbiota in this area. In this study, we utilized metagenomic technology to examine the gut microbiota of *M. himalayana* in the Sanjiangyuan National Nature Reserve on the Qinghai-Xizang Plateau. We analyzed the diversity and composition of the gut microbiota, identifying the primary microbial species. Our findings enhance the understanding of species adaptation mechanisms to extreme environments and provide valuable information for biodiversity conservation, ecological restoration, and human health. Additionally, this study proposed a hypothesis for further research on the relationship between plague outbreaks and the gut microbiota of *M. himalayana*.

## 2 Materials and methods

### 2.1 Sample collection

The fecal samples of *M. himalayana* were collected from 2019 to 2021 in Qinghai Sanjiangyuan National Nature Reserve, encompassing four administrative prefectures: Guoluo Prefecture (Maqin County, Maduo County, Gander County, Dari County, Banma County, Jiuzhi County), Yushu Prefecture (Yushu City, Nangqian County, Chengduo County, Zhiduo County, Zaduo County, Qumalai County), Hainan Prefecture (Xinghai County, Tongde County), and Huangnan Prefecture (Henan County).

*M. himalayana* was captured using the ring snare method. Intestinal samples of *M. himalayana* were collected in a sterile environment, placed in 2 mL sterile centrifuge tubes, and immediately transferred to a liquid nitrogen tank while the sample information was recorded. A total of 45 fecal samples were collected from the colon of *M. himalayana*, and data on the luminal microbiota were obtained through metagenomic sequencing. The cryopreservation tubes containing the fecal samples were quickly placed in a liquid nitrogen tank and transported to Beijing Novozymes Biotechnology Limited (Ltd). on dry ice for deoxyribonucleic acid (DNA) extraction and sequencing. Specific information on the source of the samples is given in Table 1.

**Table 1:** Geographical characteristics of *Marmota himalayana* sampling sites in Qinghai Sanjiangyuan National Nature Reserve (2019–2021).

Population	Region	Sample size	Group name	Longitude	Latitude	Altitude (m)
MAD	Maduo County, Guoluo Prefecture	3	Guoluo (GL)	98.850°E	34.833°N	4,600–4,700
MAQ	Maqing County, Guoluo Prefecture	3		100.367°E	34.550°N	3,900–4,000
GAD	Gangde County, Guoluo Prefecture	3		100.056°E	34.019°N	3,800–3,900
DAR	Dari County, Guoluo Prefecture	3		99.797°E	33.598°N	4,000–4,100
BAM	Banma County, Guoluo Prefecture	3		100.495°E	33.110°N	3,900–4,000
JUZ	Jiuzhi County, Guoluo Prefecture	3		100.990°E	33.846°N	3,600–3,700
YUS	Yushu City, Yushu Prefecture	3	Yushu (YS)	96.860°E	33.012°N	4,100–4,200
NAQ	Nangqian County, Yushu Prefecture	3		95.635°E	32.240°N	3,900–4,000
CHD	Chengduo County, Yushu Prefecture	3		97.111°E	33.369°N	3,900–4000
ZHD	Zhiduo County, Yushu Prefecture	3		95.935°E	33.854°N	4,100–4,200
ZAD	Zaduo County, Yushu Prefecture	3		95.622°E	32.775°N	3,900–4,000
QUM	Qumalai County, Yushu Prefecture	3		95.869°E	34.253°N	4,400–4,500
XIH	Xinghai County, Hainan Prefecture	3	Hainan (HA)	99.828°E	36.062°N	3,300–3,400
TOD	Tongde County, Hainan Prefecture	3		100.895°E	34.897°N	3,800–3,900
HEN	Henan County, Huangnan Prefecture	3	Hunan (HU)	101.582°E	34.737°N	3,500–3,600

Abbreviations: MAD, Maduo County; MAQ, Maqing County; GAD, Gande County; DAR, Dari County; BAM, Banma County; JUZ, Juzhi County; YUS, Yushu City; NAQ, Nangqian County; CHD, Chengduo County; ZHD, Zhiduo County; ZAD, Zaduo County; QUM, Qumalai County; XIH, Xinghai County; TOD, Tongde County; HEN, Henan County.

### 2.2 Experimental procedure and data analysis

#### 2.2.1 DNA library construction, quality control, and sequencing

Sampling 1 μg of genomic DNA was randomly fragmented into segments of approximately 350 bp using a Covaris ultrasonic disruptor for library construction using NEBNext® UltraDNA Library Prep Kit for Illumina (NEB, USA). The library preparation process includes end repair, A-tailing, sequencing adapter ligation, purification, and polymerase chain reaction (PCR) amplification. Following library construction, the integrity and size of the inserted fragments were assessed using advanced analytical technologies (AATI) analysis. Once the insert size met expectations, the effective library concentration was accurately quantified using quantitative-PCR (Q-PCR) (effective library concentration > 3 nmol/L[M]) to ensure the library quality. After passing library inspection, different libraries were pooled based on their effective concentrations and target data output requirements, and then subjected to PE150 sequencing.

#### 2.2.2 Fecal DNA extraction

DNA purity and integrity were assessed using 1 % agarose gel electrophoresis (AGE), while DNA quantification was performed with the Qubit® dsDNA Assay Kit on a Qubit® 2.0 Fluorometer (Life Technologies, CA, USA). Added 0.5 μL of sample to a centrifuge tube and diluted with sterile water until the optical density (OD) value ranged between 1.8 and 2.0.

#### 2.2.3 DNA sequencing

For library preparation, 1 μg of genomic DNA was fragmented to approximately 350 bp using a Covaris ultrasonic disruptor. The NEBNext® Ultra DNA Library Prep Kit for Illumina (NEB, USA) was used for end repair, A-tailing, adapter ligation, purification, and PCR amplification. The integrity and fragment size of the library were assessed using AATI analysis, and library concentration was quantified by Q-PCR (effective concentration > 3 nM). After quality control, libraries were pooled based on concentration and sequencing requirements, followed by PE150 sequencing.

#### 2.2.4 Bioinformatics analysis pipeline

The Fastp software (https://github.com/OpenGene/fastp) was selected for raw data preprocessing [14], while Bowtie2 (http://bowtie-bio.sourceforge.net/bowtie2/index.shtml) was employed for data alignment and removal of host-derived sequences [15]. In this study, the percentage of clean data relative to raw data ranged from 99.72 % to 99.97 %. MEGAHIT was utilized for metagenome assembly due to its efficient handling of large-scale sequencing data with low memory usage, making it highly suitable for analyzing complex microbial communities [16].

#### 2.2.5 Gene prediction and abundance analysis

MetaGeneMark was chosen for open reading frame (ORF) prediction using default parameters, as it is optimized explicitly for prokaryotic gene prediction and is widely used in metagenomic studies [17]. Genes shorter than 100 nucleotides were excluded from further analysis to remove potential sequencing artifacts [18,19]. For ORF prediction, CD-HIT (https://www.bioinformatics.org/cd-hit/) software was used to remove redundant sequences and generate a non-redundant initial gene catalog [20]. Bowtie2 was used to align the clean data of each sample to the initial gene catalog, calculating the number of reads mapping to each gene per sample. Genes with ≤ 2 reads in all samples were filtered out to determine the final gene catalog for subsequent analysis [21].

Finally, based on the number of mapped reads and gene lengths, the abundance of each gene in each sample was calculated using the following formula:

(1) $$G_{k}=\frac{r_{k}}{L_{k}}\cdot \frac{1}{\sum_{i=1}^{n}\frac{r_{i}}{L_{i}}}$$

where *r* represents the number of reads mapped to the gene, and *L* represents the gene length.

Subsequently, species composition analysis was conducted based on the abundance of each gene in each sample from the gene catalogue.

### 2.3 Species annotation

DIAMOND software (https://github.com/bbuchfink/diamond/) was used to align unigene sequences to the Micro_NR database, which contains sequences from bacteria, fungi, archaea, and viruses extracted from the National Center for Biotechnology Information (NCBI) non-redundant proteins (NR) database (https://www.ncbi.nlm.nih.gov/). The alignment was performed using the basic local alignment search tool protein (BLASTP) algorithm with an *e*-value threshold of 1×*e*^-5^ [22].

For each sequence, alignment hits with an *e*-value no more extraordinary than ten times the minimum *e*-value were selected for taxonomic classification. The lowest common ancestor (LCA) algorithm, implemented in the MEGAN software, was used to determine the taxonomic annotation of each sequence [23].

The abundance of each sample at different taxonomic levels (kingdom, phylum, class, order, family, genus, or species) was determined using the LCA annotation results and the gene abundance. Species abundance within a sample is determined by summing the abundance values of genes annotated to that species [24]. The species' gene count within a sample is quantified by the number of expressed genes (those with non-zero abundance) annotated to that species [25]. Based on abundance tables stratified by taxonomic level, Krona analysis was performed to visualize relative abundance distributions and generate abundance clustering heatmaps [26]. Intergroup alpha diversity comparisons were conducted using two-tailed Wilcoxon rank-sum tests. Ordination analyses included principal component analysis (PCA) and principal coordinate analysis (PCoA) based on Bray-Curtis distance. Differentially abundant taxa were identified through Metastat multivariate statistics.

The Metastats analysis first performs hypothesis testing on species abundance data between groups to obtain *P*-values. These *P*-values are then corrected to obtain *q*-values. Species with significant differences are identified based on the *q*-values, and box plots depicting the abundance distribution of these differentially abundant species across groups are generated. The *q*-values are obtained through false discovery rate (FDR) correction.

#### 2.3.1 Common functional database annotations

Sequence alignment of unigenes against functional databases was performed using DIAMOND software. The functional annotations were derived from Kyoto encyclopedia of genes and genomes (KEGG) (https://www.kegg.jp/kegg/) [27], evolutionary genealogy of genes: non-supervised orthologous groups (eggNOG) (https://eggnogdb.embl.de/#/app/home) [28], and carbohydrate-active enzymes (CAZy) (https://www.cazy.org/) [29] databases. After alignment, the best-matching sequences with the highest Basic Local Alignment Search Tool (BLAST) scores were selected for functional annotation [30,31]. Relative abundance at each functional level was calculated based on the alignment results [32].

#### 2.3.2 Antimicrobial resistance gene annotations

Resistance gene identification was performed by aligning unigenes against the comprehensive antibiotic resistance database (CARD) [33]. The CARD database and RGI software were utilized to identify resistance genes, as CARD provides a curated and up-to-date reference for antibiotic resistance genes, and RGI allows accurate classification of resistance mechanisms [34].

The relative abundance of each antibiotic resistance ontology (ARO) term was calculated by integrating RGI alignment results with unigene abundance data. Subsequent analyses comprised: (1) abundance visualization through histograms, hierarchical clustering heatmaps, and circular distribution plots; (2) differential ARO abundance analysis between groups; and (3) attribution analysis correlating resistance mechanisms with specific resistance genes and microbial species.

## 3 Results

### 3.1 Analysis of gut microbiota composition of *M. himalayana* in Sanjiangyuan National Nature Reserve

#### 3.1.1 Characteristics of gut microbiota of *M. himalayana*

The *M. himalayana* specimens from Sanjiangyuan Nature Reserve were divided into four groups based on administrative regions: Guoluo (GL), Yushu (YS), Hainan (HA), and Huangnan (HU). The gut microbiota of these groups shared a total of 794,860 genes (Fig. S1). Group-specific genes were as follows: GL − 265,006, HA − 70,422, HU − 33,901, and YS − 222,318.

#### 3.1.2 Differences in gut microbiota of *M. himalayana* in different regions

The microbial community in *M. himalayana* revealed that bacteria dominated, accounting for 82.87 % ± 2.94 % of the relative abundance, while archaea, viruses, and eukaryota showed much lower abundances (0.53 % ± 0.43 %, 0.20 % ± 0.13 %, and 0.04 % ± 0.18 %, respectively). Approximately 16.36 % ± 2.97 % of the sequences remained unclassified. We created a pie chart (Fig. S2) to represent the distribution of microorganisms across all samples visually.

Geographic variation in the gut microbiota of *M. himalayana* is likely influenced by factors such as diet, habitat conditions, and interspecies interactions. Stacked histograms were created to visualize the relative abundance at the phylum and genus levels (Fig. S3), showing the distribution of the top 10 bacteria in each group. At the phylum level, *Firmicutes*, *Bacteroidota*, and *Proteobacteria* were the most abundant bacterial groups, representing 74.16 % of the community. *Firmicutes* had the highest relative abundance at 40.10 % ± 6.15 %, followed by *Bacteroidota* (31.73 % ± 4.86 %) and *Proteobacteria* (2.33 % ± 0.46 %). Other phyla included *Actinobacteria* (1.06 % ± 0.29 %), *Verrucomicrobia* (0.78 % ± 0.19 %), and *Euryarchaeota* (0.53 % ± 0.43 %). Notably, 22.47 % ± 3.10 % of sequences could not be taxonomically classified, suggesting the presence of novel microorganisms and highlighting gaps in our understanding of microbial diversity.

At the genus level, 69.94 % ± 0.8 % of the sequences could not be identified. Among the recognized genera, the top 9 were *Alistipes* (11.86 % ± 1.56 %), *Bacteroides* (6.68 % ± 0.95 %), *Clostridium* (4.92 % ± 1.04 %), *Eubacterium* (3.44 % ± 0.64 %), *Lactobacillus* (2.04 % ± 1.11 %), *Pseudomonas* (0.56 % ± 0.56 %), *Chlamydia* (0.51 % ± 0.44 %), *Streptococcus* (0.065 % ± 0.02 %), and *Rhizophagus* (0.002 % ± 0.0012 %). The gut microbiota of *M. himalayana* exhibited high taxonomic diversity, with *Ehrlichia*, *Bacteroides*, *Clostridium*, and *Eubacterium* as the predominant genera, which played key roles in the host's intestinal ecosystem.

### 3.2 Alpha and Beta diversity analysis of gut microbiota of *M. himalayana*

**Fig. 1:**
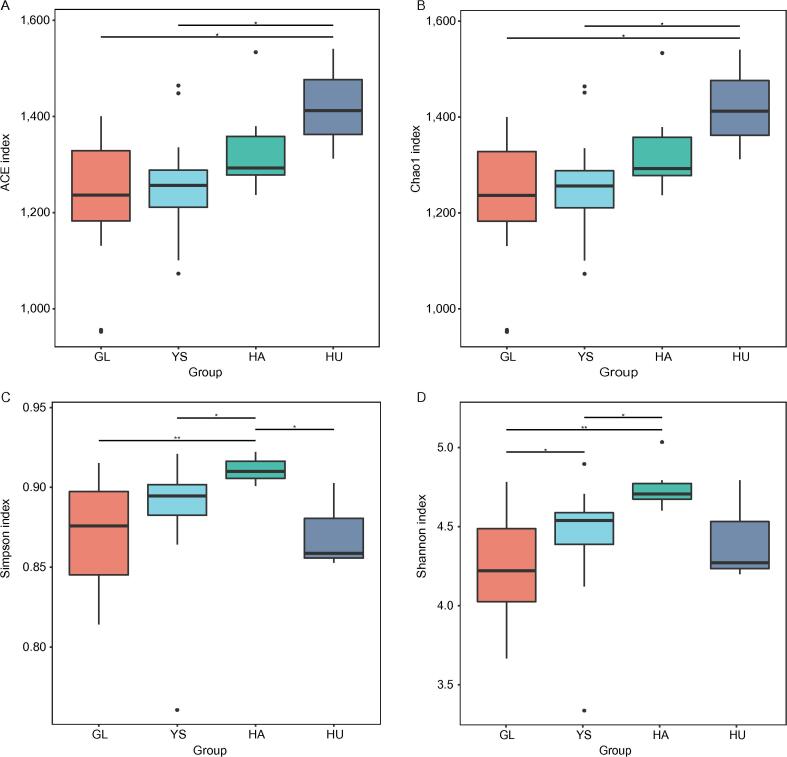
Alpha diversity indices of the gut microbiota of *M. himalayana* from different groups. *, *P* < 0.05, **, *P* < 0.01. Abbreviations: *M. himalayana, Marmota himalayana;* GL, Guoluo Prefecture; YS, Yushu prefecture; HA, Hainan Prefecture; HU, Huangnan Prefecture; ACE, abundance-based coverage estimator.

Alpha diversity analysis was conducted on the gut microbiota of four different groups of Himalayan marmots, focusing on the operational taxonomic unit (OTU) level and indices such as bundance-based coverage estimator (ACE), Chao1, Simpson, and Shannon. The results showed that at the genus level, the ACE, Chao1 values of the HU group were higher than those of the GL, YS, and HA groups, indicating that the intestinal microbial richness of the HU group was the highest (Fig. 1 A–B). The Simpson and Shannon index values of the HA group were the highest, suggesting that the microbial evenness of the HA group was relatively greater (Fig. 1 C–D).

**Fig. 2:**
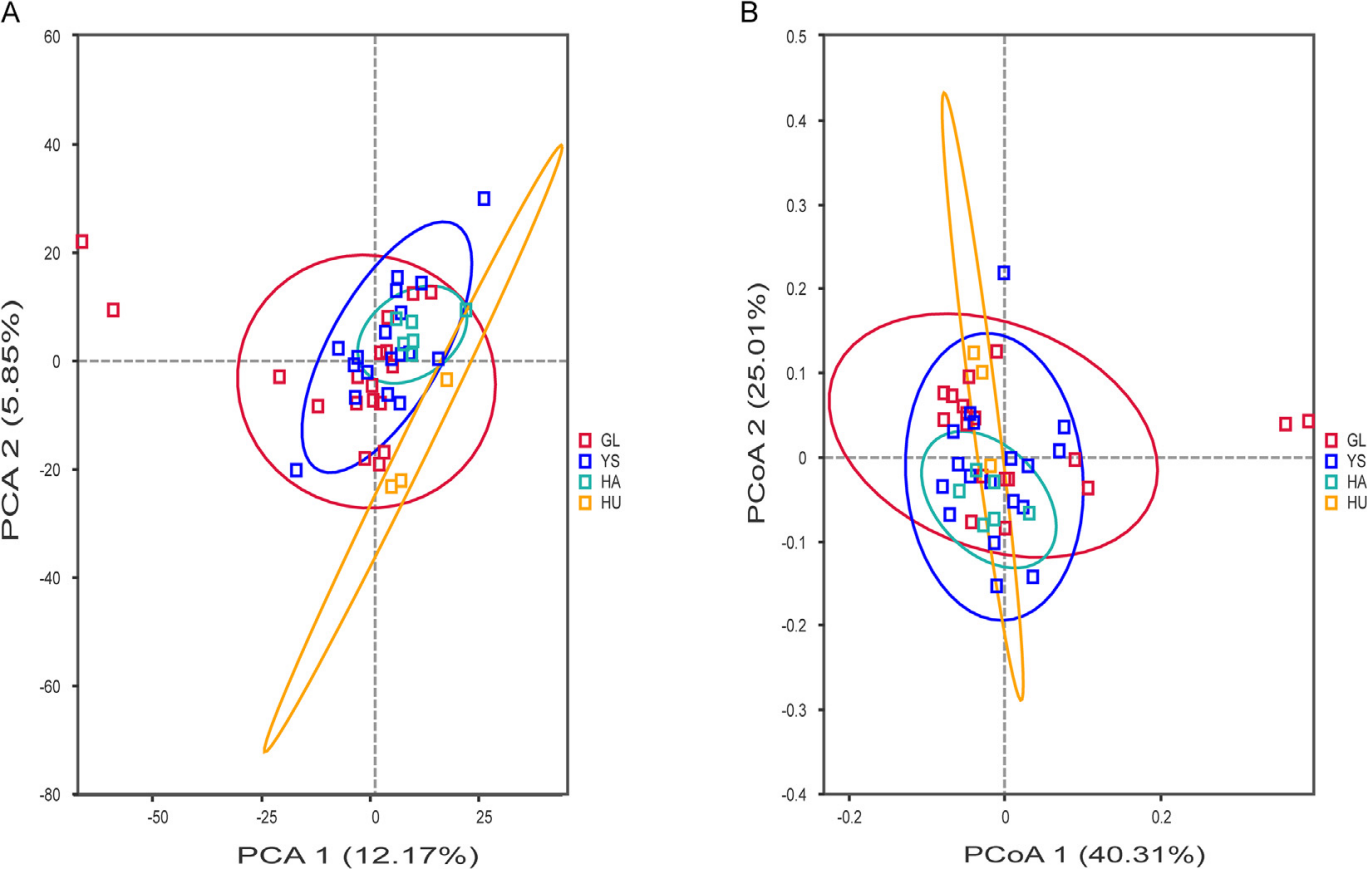
PCA (A) and PCoA (B) analysis of the gut microbiota of *M. himalayana* from different groups. Abbreviations: *M. himalayana*, *Marmota himalayana*; GL, Guoluo Prefecture; YS, Yushu Prefecture; HA, Hainan Prefecture; HU, Huangnan Prefecture; PCA, principal component analysis; PCoA, principal coordinate analysis.

Beta diversity analysis was employed to examine the variations in gut microbiota composition of *M. himalayana* across distinct research regions (Fig. 2). The results revealed that while four groups exhibited some clustering, complete segregation was not observed. This indicates that geographic region exerts a moderate influence on the gut microbiota composition of *M. himalayana*.

### 3.3 Annotation results of functional genes of gut microbiota of *M. himalayana*

#### 3.3.1 Functional annotation and differential analysis in gut microbiota of M. himalayana based on the KEGG database

**Fig. 3:**
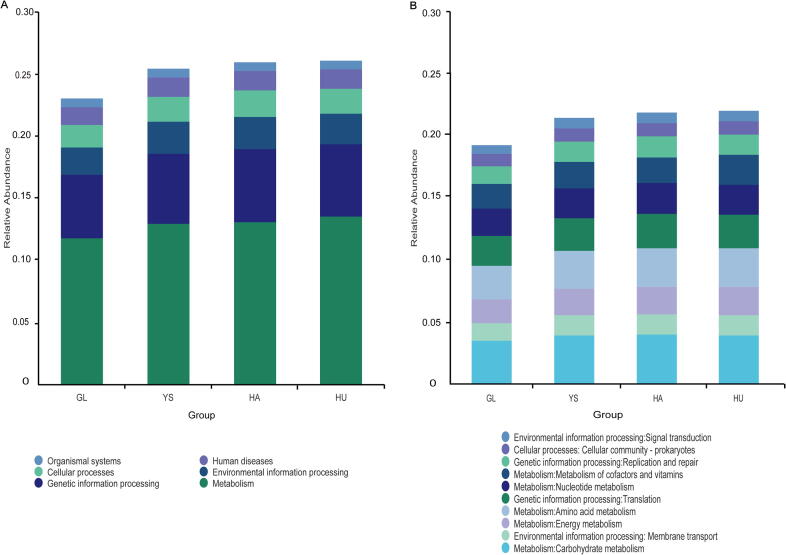
Stacked bar chart of functional gene annotation of *M. himalayana* at level 1 (A) and level 2 (B) levels based on the KEGG database. Abbreviations: *M. himalayana*, *Marmota himalayana*; GL, Guoluo Prefecture; YS, Yushu Prefecture; HA, Hainan Prefecture; HU, Huangnan Prefecture; KEGG, Kyoto encyclopedia of genes and genomes.

Functional gene annotation *via* the KEGG database indicated that metabolic pathways were the most abundant level-1 (Fig. 3A) functional category in the gut microbiota of all sampled *M. himalayana*, accounting for over 12.0 % of annotated functions, which were followed by GIP and EIP, with organismal systems (OS) being the least abundant. At level 2 (Fig. 3B), carbohydrate metabolism was the most prevalent function (over 3.6 %), followed by amino acid metabolism and translation, while signal transduction had the lowest percentage. These results suggest that *M. himalayana*'s gut microbiota exhibits high metabolic activity, especially in carbohydrate metabolism, which is crucial for the host's energy acquisition and nutrient utilization. The increased abundance of genes related to environmental and genetic information processing indicates that these microbial communities play significant roles in environmental adaptation and genetic variation. The lower relative abundance of biological systems pathways implies a limited contribution of the microbiota to complex biological structures and regulatory functions.

At KEGG level 2, notable functional differences were observed among the geographically distinct populations of *M. himalayana*. The GL group exhibited higher relative abundance in pathways associated with viral infectious diseases, digestive functions, and immune system processes, suggesting increased pathogen exposure or enhanced immune responses in this population. In contrast, the HU group showed abundant metabolic functions related to cellular transport and catabolism, glycan biosynthesis, and metabolism, followed by terpenoid and polyketide metabolism, reflecting higher energy acquisition and nutrient processing demands. The YS group had a greater abundance of transport and signal transduction functions within environmental information processing. The HA group demonstrated the highest abundance in the endocrine system within OS, followed by endocrine and metabolic diseases in human diseases and environmental adaptation in OS. These profiles likely reflect population-specific adaptations to different ecological niches.

Significant differences between the GL and HU groups were found in three functional categories: nervous system signaling, viral pathogen response, and energy metabolism, suggesting distinct regulatory mechanisms in these areas. The YS and HA groups showed significant differences in pathways related to endocrine-metabolic disorders, transcription, cancers, nucleotide metabolism, aging, and translation, indicating divergent microbial strategies for modulating host metabolism, cellular homeostasis, and disease susceptibility. Additionally, comparative analysis between HA and HU groups revealed significant differences in endocrine and metabolic diseases, eukaryotic cell communities, and viral infectious diseases, while other pathways showed no significant variation.

#### 3.3.2 EggNOG-based functional annotation and differential analysis in the gut microbiota of M. himalayana

EggNOG database functional annotation revealed low overall abundance across level 1 functional categories in the gut microbiota of *M. himalayana*, with uncharacterized functions accounting for the highest relative abundance (20.00 %), highlighting the current limitations in functional annotation of gut microbiota, as many microbial genes remain uncharacterized. The second most abundant category was DNA replication, recombination, and repair (7.50 %), indicating robust genomic maintenance and DNA damage response capabilities. At level 2, ATP-binding cassette (ABC) transporters were the most abundant category (1.51 %), underlining their essential role in nutrient acquisition and metabolite efflux, critical for microbial survival and metabolism. The second most abundant category, transposases (1.25 %), is involved in genomic recombination and microbial genome evolution.

**Fig. 4:**
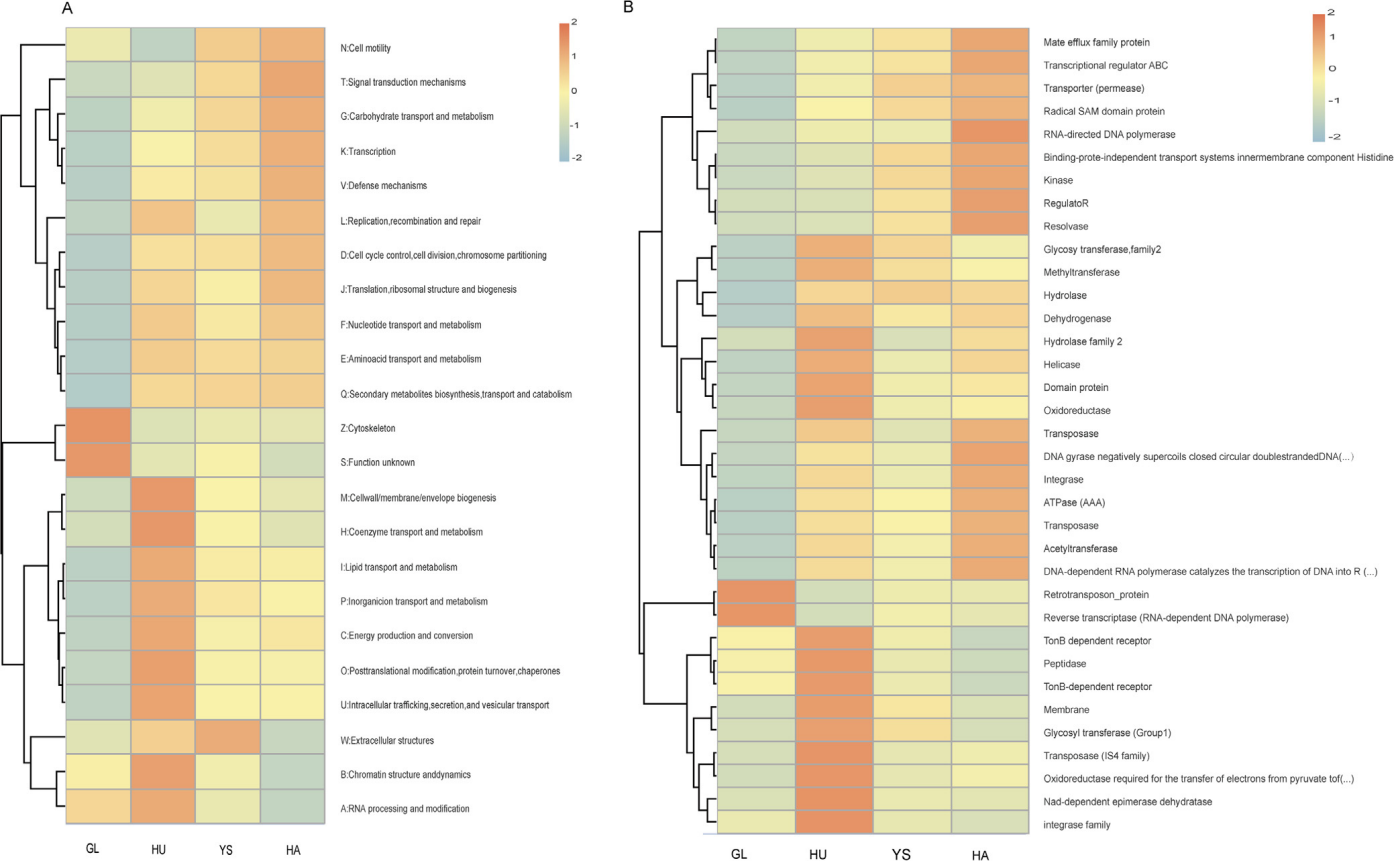
Heat map of functional abundance clustering of *M. himalayana* at level 1 (A) and level 2 (B) levels based on eggNOG database. Abbreviations: *M. himalayana*, *Marmota himalayana*; GL, Guoluo Prefecture; YS, Yushu Prefecture; HA, Hainan Prefecture; HU, Huangnan Prefecture; eggNOG, non-supervised orthologous groups.

Clustering analysis based on the eggNOG database at level 1 (Fig. 4A) showed distinct functional clusters across different groups. The GL group exhibited prominent functional clusters related to cytoskeleton functions and some uncharacterized functions, suggesting that the microbiota in this group plays a role in cell morphology, motility, and division. The YS group exhibited abundant functional clusters related to extracellular structures, including flagella, pili, and cell walls, which mediate host-microbe and microbe-microbe interactions. The HA group exhibited enhanced functional clusters related to signal transduction, carbohydrate transport, metabolism, transcription, and defense mechanisms, reflecting environmental sensing and stress response capabilities. The HU group was characterized by abundant clusters associated with wall/membrane/envelope biogenesis, coenzyme transport, and metabolism, indicating complex cellular architecture and energy metabolism regulatory networks.

Comparative analysis at the eggNOG level 2 (Fig. 4B) revealed significant differences among populations of homologous protein family abundance. The GL group had abundant retrotransposon proteins and ribonucleic acid (RNA)-dependent DNA polymerase, reflecting retrotransposition events mediated by reverse transcriptase activity. The YS group showed high protein diversity with an even distribution across protein families. The HA group exhibited abundant RNA-directed DNA polymerase, kinases, and DNA gyrases, which are crucial for DNA replication, repair, and chromosomal stability. The HU group displayed the highest diversity and abundance of homologous protein families, reflecting complex adaptations to the environment and host.

A Metastats analysis based on the eggNOG database was conducted to assess the functional characteristics of the gut microbiota in *M. himalayana* from different regional environments at level 1. The Analysis revealed significant functional differences across the groups. Notably, 13 functions differed significantly between the GL and HA groups, 7 between the HA and HU groups, and 6 between the GL and HU groups. However, no significant differences in functional abundance were observed between the YS and GL, HU, or HA groups.

#### 3.3.3 Cazy-based functional annotation and differential analysis in the gut microbiota of M. himalayana

**Fig. 5:**
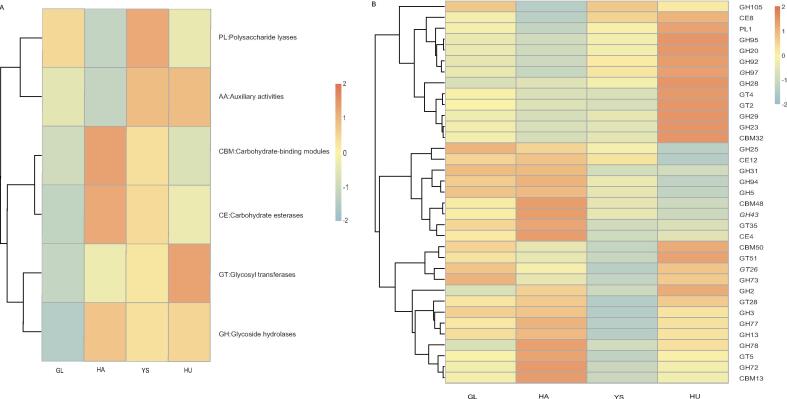
Functional abundance clustering heat map of *M. himalayana* at level 1 (A) and level 2 (B) based on the carbohydrate-active enzymes database. Abbreviations: *M. himalayana*, *Marmota himalayana*; GL, Guoluo Prefecture; YS, Yushu Prefecture; HA, Hainan Prefecture; HU, Huangnan Prefecture; CAZy, carbohydrate-active enzymes.

To investigate the clustering differences of carbohydrate enzymes in the gut microbiota of *M. himalayana* in the Sanjiangyuan National Nature Reserve, Qinghai, the top 35 carbohydrate enzymes by abundance were selected for clustering heatmap analysis at both level 1 and level 2 (Fig. 5).

At level 1 (Fig. 5A), no significant differences in carbohydrate enzyme clusters were observed in the GL group. However, the YS group exhibited a higher abundance of polysaccharide lyases (PLs), indicating enhanced capabilities for polysaccharide degradation and utilization in their gut microbial community. The HA group displayed elevated abundances of carbohydrate-binding modules (CBMs) and carbohydrate esterases (CEs), reflecting efficient carbohydrate metabolism and resource utilization. The HU group showed a dominant abundance of glycosyl transferases (GTs), suggesting an increased capacity for synthesizing and modifying complex carbohydrates in their gut microbiota. At level 2 (Fig. 5B), the GL group exhibited the highest abundance of carbohydrate enzyme clusters characterized as GH105. In contrast, the YS group had higher GH25, GH31, and GH73 abundances. The HA group predominantly contained clusters CBM48, GH43, GT35, CE4, GH78, GH72, and CBM13, while the HU group showed higher abundances of GH95, GH20, GH92, GH97, GH28, GT4, GT2, GH29, GH23, and CBM32 clusters.

A Metastats analysis was conducted to assess the differences in the abundance of carbohydrate-active enzymes in the gut microbiota of *M. himalayana* at level 1. In the CBM category, the HA group exhibited significantly higher abundance compared to the HU group (*P* = 0.003), and the YS group also showed significantly higher abundance than the HU group (*P* = 0.043). These findings suggest that the HA and YS groups may have enhanced capabilities in specific carbohydrate recognition, binding, and metabolic processing in their gut microbiota. In the auxiliary activities (AA) category, the GL group showed significantly higher abundance than the HA group (*P* = 0.038), indicating that the GL group's gut microbiota has a greater capacity for complex carbohydrate modification and degradation. No significant differences in the abundance of carbohydrate-active enzymes were observed among the other groups, indicating similar functional capabilities in their microbial communities.

### 3.4 Annotation of resistance genes

#### 3.4.1 Abundance profile of resistance genes

The content and percentage of ARO in *M. himalayana* samples from different regions were calculated based on the relative abundance of resistance genes. The top 20 AROs ranked by abundance were selected. Among all the samples, the top ten resistance genes, listed in descending order of abundance, were as follows: *MCR-1.2* (9.914 parts per million [ppm], 2.963 %), *SHV-100* (9.016 ppm, 2.925 %), *MexH* (9.537 ppm, 2.868 %), *vanC* (9.122 ppm, 2.648 %), *OXA-184* (6.459 ppm,1.950 %), *vanM* (5.251 ppm, 1.563 %), *vanRO* (3.416 ppm, 1.028 %), *AAC3-IIIa* (3.362 ppm, 0.968 %), *mefC* (2.581 ppm, 0.745 %), *vanTG* (1.666 ppm, 0.503 %).

Significant differences in ARO abundance profiles were observed among different groups in data analysis, with the relative abundance of AROs presented in the table, respectively (Table 2), reflecting variations in ecological pressures and historical antibiotic exposure. The GL group exhibited the highest abundance of the *vanC* resistance gene (24.454 ppm, 6.540 %), suggesting enhanced resistance to glycopeptide antibiotics in this region. The highest abundance of the *SHV-100* β-lactamase gene in the HA (10.436 ppm, 4.140 %), YS (9.419 ppm, 2.970 %), and HU (8.424 ppm, 2.510 %) populations suggests widespread resistance to β-lactam antibiotics in these regions.

**Table 2:** ARO abundance profiles in the gut microbiota of *M. himalayana* from different groups.

Group	GL		YS		HA		HU	
	Mean	Percentage (%)	Mean	Percentage (%)	Mean	Percentage (%)	Mean	Percentage (%)
Vancomycin resistance gene cluster C (*vanC*)	24.454	6.54	7.282	2.30	3.369	1.34	1.382	0.41
Multidrug efflux pump membrane fusion protein H (*MexH*)	22.573	6.04	7.722	2.43	6.713	2.66	1.138	0.34
Vancomycin resistance-associated protein TG (vanTG)	4.527	1.21	0.072	0.02	1.686	0.67	0.379	0.11
Multidrug colistin resistance-1.2 (*MCR-1.2*)	23.358	6.25	8.382	2.64	6.104	2.42	1.813	0.54
Aminoglycoside phosphotransferase 4-Ib (*APH4-I*)	0.107	0.03	0.205	0.06	1.316	0.52	0.664	0.20
Tetracycline resistance gene Q (*tetQ*)	0.013	0.00	0.000	0.00	0.379	0.15	0.00	0.00
Tetracycline resistance gene A (*TetA*)	0.270	0.07	0.000	0.00	0.000	0.00	0.00	0.00
Vancomycin resistance gene cluster M (*vanM*)	13.161	3.52	4.690	1.48	3.151	1.25	0.00	0.00
OXA-184 β-lactamase (*OXA-184*)	15.083	4.03	5.384	1.70	4.737	1.88	0.632	0.19
Staphylococcus aureus mupA gene conferring high-level mupirocin resistance (Staphylococcus_mupA_conferring)	0.750	0.20	0.408	0.13	2.386	0.95	0.579	0.17
*vanRO*	7.690	2.06	2.995	0.94	2.234	0.89	0.746	0.22
Aminoglycoside acetyltransferase 3-IIIa (*AAC3-IIIa*)	9.578	2.56	2.754	0.87	1.114	0.44	0.00	0.00
Macrolide efflux gene C (*mefC*)	7.460	2.00	1.925	0.61	0.938	0.37	0.00	0.00
Polyamine modulator of resistance E (*PmrE*)	0.198	0.05	1.231	0.39	0.053	0.02	2.326	0.69
Outer membrane protein Z (*OprZ*)	9.295	2.49	2.466	0.78	0.418	0.17	0.00	0.00
SHV-type extended-spectrum β-Lactamase 100 (*SHV-100*)	7.786	2.08	9.419	2.97	10.436	4.14	8.424	2.51
Vancomycin resistance gene WI (*vanWI*)	0.064	0.02	0.284	0.09	0.811	0.32	0.00	0.00

Abbreviations: *M. himalayana*, *Marmota himalayana*; GL, Guoluo Prefecture; YS, Yushu prefecture; HA, Hainan Prefecture; HU, Huangnan Prefecture; ARO, antibiotic resistance ontology.

#### 3.4.2 Distribution of resistance gene types and cluster analysis

**Fig. 6:**
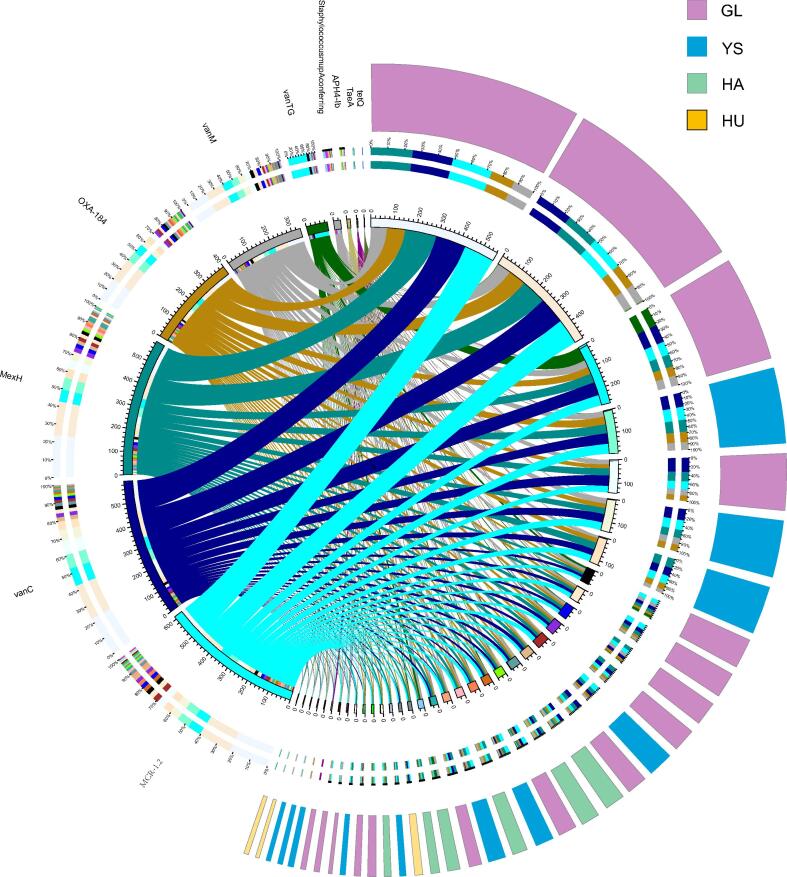
The overview circle diagram of resistance genes in different groups with high abundance. Abbreviations: *M. himalayana*, *Marmota himalayana*; GL, Guoluo Prefecture; YS, Yushu Prefecture; HA, Hainan Prefecture; HU, Huangnan Prefecture.

The annotation of antibiotic resistance genes (ARGs) from the CARD provided valuable insights into resistance mechanisms across different *M. himalayana* populations. The overview circle diagram (Fig. 6) presented the relative abundance of resistance genes in samples with high abundance, providing a clear visualization of the distribution of ARGs within the microbiota of different regional populations. It was revealed through analysis that the *MexH*, *vanC*, and *MCR-1.2* resistance genes were the most clustered and exhibited the highest relative abundance in the GL group, suggesting the potential influence of specific environmental or ecological factors contributing to this increased abundance. The *MexH* gene is associated with resistance to multiple antibiotics, including macrolides, while *vanC* confers resistance to glycopeptides such as vancomycin, and *MCR-1.2* is linked to resistance to polymyxins, including colistin. The clustering of these resistance genes highlights the potential for selective pressure or environmental factors in the Guoluo regions that may influence the distribution of these resistance mechanisms in the *M. himalayana* gut microbiota.

**Fig. 7:**
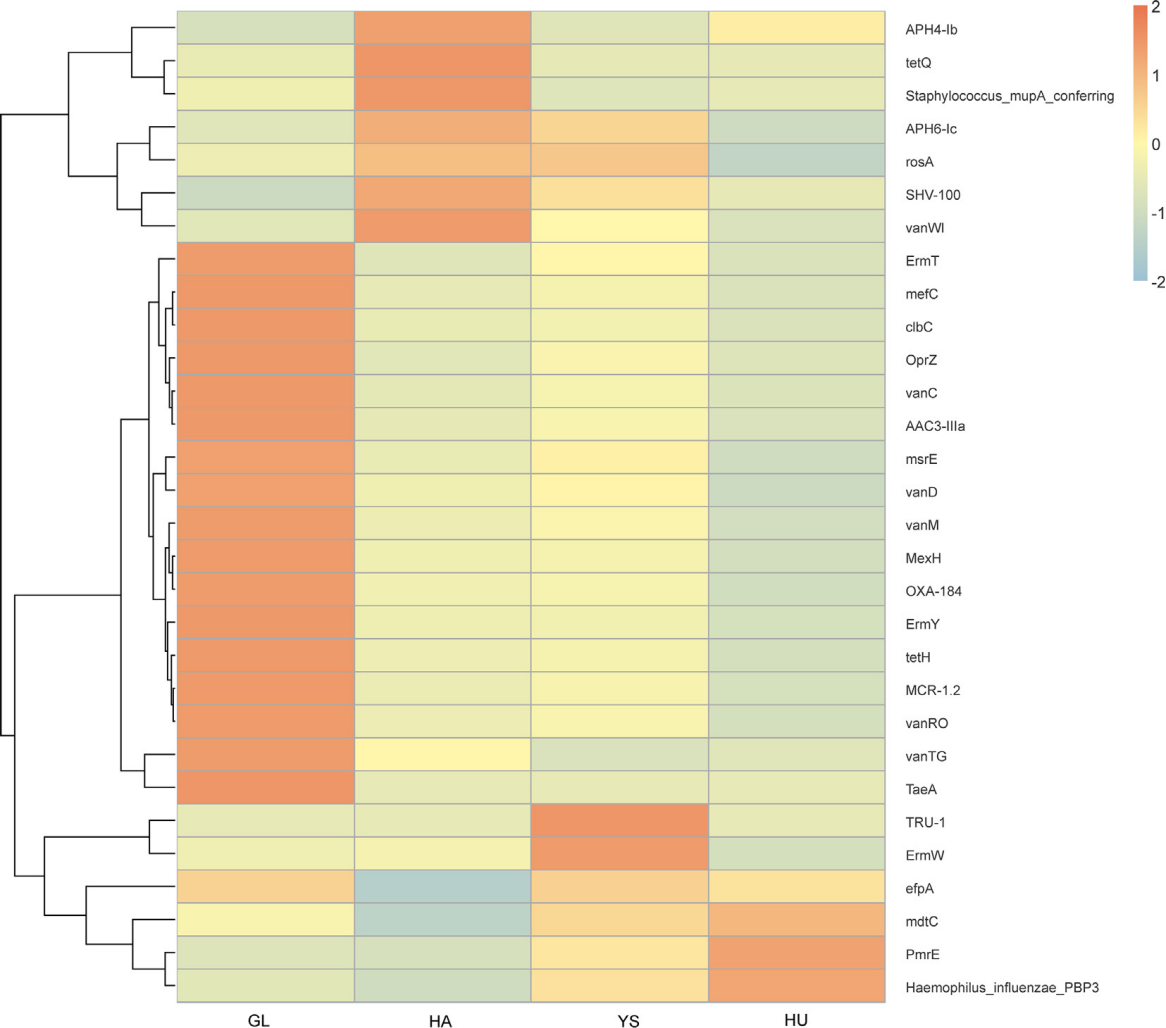
Heat map of ARO abundance clustering. Abbreviations: ARO, antibiotic resistance ontology; *M. himalayana*, *Marmota himalayana*; GL, Guoluo Prefecture; YS, Yushu Prefecture; HA, Hainan Prefecture; HU, Huangnan Prefecture.

The ARO abundance clustering heatmap (Fig. 7) revealed that the GL group exhibited significantly higher clustered ARO abundance compared to the other groups for several AROs, including *ErmT*, *mefC*, *clbC*, *OprZ*, *vanC*, *AAC3 − IIIa*, *msrE*, *vanD*, *vanM*, *MexH*, *OXA-184*, *ErmY*, *tetH*, *MCR-1.2*, *vanRO*, *vanTG*, and *TetA*. The GL population exhibited a significantly higher abundance of clustered antibiotic resistance genes, particularly those conferring resistance to vancomycin and colistin, than other populations. The YS group exhibited a significantly higher abundance of *TRU-1* and *ErmW* resistance genes, suggesting prevalent resistance to macrolide and aminoglycoside antibiotics. *APH4 − Ib*, *tetQ*, Staphylococcus_mupA_conferring, and *vanWI* showed significantly higher aggregated ARO abundance than the other groups in the HA groups. These resistance genes confer protection against tetracyclines, aminoglycosides, and β-lactam antibiotics. The HU group exhibited significantly higher aggregated ARO abundance for PmrE and Haemophilus_influenzae_PBP3, which encode proteins involved in antimicrobial resistance mechanisms.

#### 3.4.3 Analysis of differences between groups of resistance gene types

The top 20 ARGs were ranked by abundance, as shown in Fig. S4, under the Metastats analysis. The results revealed notable differences in the resistance gene profiles between different *M. himalayana* populations. Specifically, the HA and HU groups exhibited significantly higher levels of *IMP_12*, *hp1181*, and *tetB60* than the GL and YS groups. These genes are associated with resistance to carbapenem and tetracycline antibiotics, suggesting that the microflora in the HA and HU groups possess robust resistance mechanisms against these classes of antibiotics. On the other hand, the YS group showed a significantly lower abundance of the *bcrA* and *vgbA* genes, which are related to multidrug efflux mechanisms, suggesting that the gut microbiota of the YS group may have reduced capacity for effluxing multiple antibiotics, leading to a lower overall resistance profile.

### 3.5 Analysis of human plague, animal epidemic situations, and *Y. Pestis* genotyping correlation in the natural plague foci of the Sanjiangyuan Nature Reserve

We collected relevant information on human and animal plague cases [35,36], as well as genotypic data of *Y. pestis*, which includes 245 strains identified by DFR [11] and 78 strains identified by single nucleotide polymorphism (SNP) [37], in the Sanjiangyuan plague focus from Qinghai Province from 1954 to 2022. A total of 90 human plague outbreaks were recorded, involving 253 patients and resulting in 153 fatalities, which corresponds to a case-fatality rate of 60.47 % in the Sanjiangyuan region. The primary sources of infection include 53 cases from skinning and consuming *M. himalayana*, 15 cases from flea bites, 14 cases from skinning and consuming Tibetan sheep, seven cases resulting from contact with other infected animals, and one case with an unknown source. There were 293 animal plague outbreaks, with 240 originating from *M. himalayana*. The YS group had the highest incidence of human plague cases and animal plague occurrences, along with the most significant number of unique genetic types of *Y. pestis* (such as types 10, 14, 30, and 36). In contrast, the other three groups experienced fewer plague outbreaks, with the genetic types of the strains predominantly being types 5 or 8, suggesting a correlation between the occurrence of plague, animal populations, and the unique genetic types of *Y. pestis*. The statistical results are presented in Table 3.

**Table 3:** Statistical results of human plague, animal plague, and *Y. pestis* genotyping in Qinghai Sanjiangyuan National Nature Reserve (1954–2022).

Group	Human plague								Animal plague			Genetic typing of *Y. pestis*	
	Number of occurrences	Source				Number of cases	Number of deaths	Case fatality rate (%)	Total	Source		DFR genotypes (number of strains)	SNP genotypes (number of strains)
		*M. himalayana*	Flea	Other animals	Unknown					*M. himalayana*	Vector		
GL	11	6	1	3	1	30	20	66.67	8	6	2	5 (3), 8 (7)	1.IN (8)
YS	72	43	14	15	0	180	113	60.43	263	220	43	5 (143), 8 (3), 10 (8), 14 (11), 30 (2), 36 (47)	1.IN (58), 0.PE (3), 2.ANT (1)
HA	5	3	0	2	0	35	12	34.29	22	14	8	8 (19), 21 (1)	1.IN (6), 0.PE (1)
HU	2	1	0	1	0	8	8	100.00	0	0	0	5 (1)	1.IN (1)

Abbreviations: *M. himalayana*, *Marmota himalayana*; *Y. pestis*, *Yersinia pestis*; GL, Guoluo Prefecture; YS, Yushu prefecture; HA, Hainan Prefecture; HU, Huangnan Prefecture; DFR, different region; SNP, single nucleotide polymorphism.

A total of 90 human plague outbreaks were recorded, involving 253 patients and resulting in 153 fatalities, which corresponds to a case-fatality rate of 60.47 % in the Sanjiangyuan region. The primary sources of infection include 53 cases from skinning and consuming *M. himalayana*, 15 cases from flea bites, 14 cases from skinning and consuming Tibetan sheep, seven cases from contact with other infected animals, and one case with an unknown source. There were 293 animal plague outbreaks, with 240 originating from *M. himalayana*. The YS group has the highest incidence of human plague cases and animal plague occurrences, as well as the most significant number of unique genetic types of *Y. pestis* (such as types 10, 14, 30, and 36). In contrast, the other three groups experienced fewer plague outbreaks, with the genetic types of the strains predominantly being types 5 or 8, which suggested a correlation between the occurrence of plague, animal populations, and the unique genetic types of *Y. pestis*.

## 4 Discussion

The high altitude and harsh environment of the Qinghai-Xizang Plateau pose numerous challenges for herbivores, including food shortages due to the short growing season of plants. Therefore, studying the diversity and function of gut microbiota can help monitor and analyze the current status of host survival and adaptation mechanisms. *M. himalayana*, positioned in the middle of the food chain within the grassland ecosystem, serves as both predator and prey, influencing the development of the ecosystem. The stability of its gut microbiota is vital for maintaining the stability of the grassland ecosystem. Our study analyzes the gut microbiota structure of *M. himalayana*, providing a comprehensive understanding of *M. himalayana* from various perspectives, including dietary structure, host, and physiological changes, and comparative analyses of the abundance, composition, and differences of gut microbiota in *M. himalayana* from different regions. This analysis is essential for understanding the dietary adaptations of the hosts, monitoring their health, and providing early warning regarding potential outbreaks of plague.

Specifically, we noted that 22.47 % of sequences remained unclassified at the phylum level. In comparison, 69.94 % remained unclassified at the genus level, which may be attributed to two main factors: First, the reference sequences for plateau-associated microorganisms in public databases such as SILVA and NCBI NR are relatively limited, representing less than 12 % of the total entries. This underrepresentation leads to frequent “unclassified” outcomes due to the lack of matching reference data [38]. Second, existing classification tools have inherent limitations in detecting and identifying novel microbial taxa, especially in extreme and poorly explored environments like the Qinghai-Xizang Plateau. For instance, the proportion of unclassified sequences at the genus level in glacier samples from the plateau can reach up to 69.94 %, significantly exceeding the typical less than 30 % observed in low-altitude ecosystems. Therefore, the high proportion of unclassified taxa likely reflects both database bias and genuine microbial novelty, highlighting the need for expanding reference databases and improving annotation tools. The complexity of the mammalian digestive tract creates a rich ecological niche for commensal microorganisms. Over long periods of evolution, animals with different diets have developed gut microbiota compatible with their diets. Herbivores possess the highest diversity of gut microbiota, followed by omnivores, with carnivores showing the least diversity [39]. While numerous studies have focused on animal intestinal tracts, there is a notable lack of research on wildlife flora, especially regarding the macrogenomic study of the gut microbiota of *M. himalayana*, a reservoir host for plague on the Qinghai-Xizang Plateau. This study is the first to characterize the fecal bacterial microbiota of *M. himalayana* using high-throughput sequencing. Our findings indicate that *M. himalayana* has similar dominant bacteria in different geographic regions, suggesting commonality in the gut microbiota of *M. himalayana* from diverse regions. This result is significant as it underscores the co-evolution of mammalian gut microbiota with their hosts. All samples in this study were collected from high-altitude areas exceeding 3,000 m above sea level, which displayed characteristics typical of plateaus, including a dominance of Firmicutes. Although Firmicutes and Bacteroidetes remained the dominant phyla at the phylum level, their total percentage was only 71.83 %, below the 88 % commonly reported in most studies [40,41], suggesting that changes in the composition and abundance of the gut microbiota community play a crucial role in environmental adaptation. In this study, Proteobacteria ranked third in abundance, and their presence was associated with nutritional deficiencies [42]. The variability of food sources and nutritional quality in the wild environment, where *M. himalayana* faced nutritional deficiencies, has likely increased Proteobacteria’s abundance [43]. The high abundance of Proteobacteria in the gastrointestinal tract of *M. himalayana* may serve as a strategy for coping with its complex diet. *M. himalayana* are herbivorous animals that ingest substantial quantities of plant cell walls, which are difficult to digest and assimilate. This study identified *Alistipes*, *Bacteroides*, and *Clostridium* as the predominant bacterial genera within the *M. himalayana*. These bacterial generaimprove the decomposition of plant cellulose, enhance carbohydrate metabolism, and increase nutrient acquisition, aiding adaptation to the marmot's high-fiber dietary habits. The extreme conditions at high altitudes, including scarce oxygen, low temperatures, and intense ultraviolet radiation, create significant survival challenges for wildlife inhabiting the Qinghai-Xizang Plateau. In response, the gut microbiota of these animals plays a crucial role as a symbiotic system, helping adaptation to the harsh environment [44]. Intestinal microorganisms degrade complex carbohydrates through the encoding of CAZymes, producing metabolites such as short-chain fatty acids (SCFAs). These microorganisms significantly influence metabolic and physiological processes, working synergistically to uphold intestinal health. Based on the KEGG database, the most abundant functions in the gut microbiota of *M. himalayana* are related to both metabolism and carbohydrate metabolism. These functions are essential for the overall functionality of the host organism [45]. Carbohydrates were the primary material basis for host energy provision and metabolic activities. The metabolic processing was crucial for energy production, growth, development, and the interconversion of various substances, and was also served as an important energy source for the host's gut microbiota [46]. The physiological process involved in the decomposition and absorption of plant-based food by *M. himalayana* demonstrated that its gut microbiota was well adapted to its dietary intake.

Results from the CAZy database annotations support these findings. Notably, the abundance of glycoside hydrolase family 43 (GH43) and glycosyl transferase family 2 (GT2) enzymes in the HU/HA groups is markedly high. These enzymes facilitate the hydrolysis of hemicellulose, cellulose, and pectin present in alpine vegetation, thereby optimizing the utilization of dietary carbohydrates. This specialized capacity to digest alpine vegetation addresses the challenge to *M. himalayana* of limited food resources in high-altitude environments.

Analysis of the eggNOG database revealed that the most abundant functions at various hierarchical levels in the gut microbiota of *M. himalayana* include DNA replication, recombination, and repair, as well as ABC transporter and transposase functions. These functional genes play crucial roles in maintaining genomic stability within the gut microbiota and microflora in response to environmental changes. ABC transporters affect the immune capacity and metabolic processes of *M. himalayana*, contributing to a balanced relationship between the gut microbiota and the host [47]. Short-chain fatty acids produced from a high-fiber diet can reduce pathogenic bacteria, decrease transposase activity, and enhance immunity and intestinal health in *M. himalayana* [48]. These findings suggest that these highly abundant functions provide marmots with dietary and metabolic flexibility, improving genomic stability within the intestinal microbial community and enhancing the marmots' adaptability to harsh environments. The fact that 20 % of eggNOG functions remain uncharacterized highlights the pronounced limitations in coverage, classification granularity, and depth of functional annotation in current functional databases of wildlife microbiomes, resulting in a higher proportion of uncharacterized functions. In summary, the gut microbiota of *M. himalayana* plays an enormous role in adapting to its diverse diet, suggesting a coevolutionary connection between the animal and its gut microbiota. It is necessary to conduct further analyses of its metabolic pathways in more detail to enhance the understanding of the intestinal microbiome function of *M. himalayana*; understanding carbohydrate enzyme function and regulation in the gut microbiota of *M. himalayana* is essential for elucidating microbial community dynamics and their impact on host health, disease susceptibility, and ecological adaptation to the plateau environment.

On the other hand, the vulnerability of plateau ecosystems may exacerbate the microbial response to extreme environments, including the accumulation of ARGs. Consequently, studying the distribution of ARGs is crucial for assessing ecological health. Currently, more than 700,000 deaths annually are due to antibiotic-resistant bacterial infections worldwide, with the majority occurring in low- and middle-income countries. Coupled with the slow pace of new antibiotic development, a future scenario without effective drugs may arise [49]. Animal intestines are identified as reservoirs of ARGs, but our knowledge of these resistance genes in animal gut microbiota remains limited [50]. Antibiotics and drug-resistant bacteria from human medical and agricultural sources leak into the environment through contaminated water and soil, spreading among wildlife. Thus, wildlife samples are critical for assessing antibiotic contamination. In this study, *M. himalayana* exhibited the highest abundance of the resistance gene *MCR-1.2*, identified by Italian researchers in 2016. This gene promotes bacterial resistance to polymyxins, the last line of defense against gram-negative, drug-resistant bacteria. The presence of *MCR-1.2* in wildlife populations, such as *M. himalayana*, reflects the broader ecological issue of antibiotic resistance, as these animals interact with and accumulate resistance genes from their contaminated environments [51]. It had been confirmed that it is a genuine problem of illicit utilization of feed additives, including antibiotics such as polymyxin, by certain agricultural and pastoral livestock operators in the Qinghai-Xizang Plateau region. This issue warrants significant societal concern regarding the development of bacterial resistance originating from animal sources. Moving forward, strict management of veterinary drugs and feed additives in the livestock industry is imperative to reduce the usage of veterinary antibiotics and prevent the residual presence of high-risk antibiotics such as polymyxin in wildlife feces and soil. Furthermore, the spread of ARGs in wildlife can significantly impact ecosystem health as wildlife populations are exposed to anthropogenic sources of antibiotic resistance through soil, water, and their diets. These organisms’ reservoirs of resistant bacteria can be transferred across species. This cross-species interaction can potentially disrupt ecological balances, alter microbial community dynamics, and affect the resilience of ecosystems. The accumulation of antibiotic-resistance genes in wildlife may reflect shifts in microbial biodiversity, which in turn can potentially influence ecological processes such as nutrient cycling, soil health, and plant-pollinator interactions [52]. In just a few years, *M. himalayana* on the Qinghai-Xizang Plateau has been found to harbor the highest abundance of gut microbiota, highlighting concerns over drug resistance due to antimicrobial misuse in China. Our study identified resistance genes, including *AAC3-IIIa*, *MexH*, *vanC*, *vanM*, *vanRO*, *vanTG*, *OXA-184*, and *SHV-100*. It is crucial to note that many pathogenic bacteria require specific conditions to cause disease [53]. The selective pressure for drug resistance in gut microbiota increases with multiple antibiotics and drugs, thereby influencing the composition and abundance of drug-resistant microbiota in animals [54,55], which complicates the treatment of diseases in wildlife.

Additionally, the impact of antibiotic residues on wildlife is more pronounced in areas with high human activity [56,57], which aligns with our findings. Moreover, the abundance of resistance genes in the gut microbiota of different *M. himalayana* populations varied, likely due to regional differences resulting in different food compositions, significantly influencing the changes in ARGs in the gut microbiota of *M. himalayana*. Future research should focus on several key areas. First, it is essential to investigate the composition and function of gut microbiota in wild animals in the Qinghai-Xizang Plateau and the functional mechanisms of potential pathogenic bacteria, which will not only help us understand the role of gut microbiota in host health but also provide important insights for disease prevention and treatment [58]. Second, given the regional differences in antimicrobial resistance gene profiles, future studies should examine these genes’ origins and transmission mechanisms in *M. himalayana* populations and their potential impacts on host health and ecosystem dynamics. This research requires interdisciplinary collaboration involving fields such as medicine, agriculture, veterinary science, and environmental studies to develop evidence-based antibiotic stewardship policies that mitigate the spread of plague in China, where the *Himalaya* is the predominant host of plague focus and plays a pivotal role in the maintenance in China, transmission, and prevalence of plague, being directly or indirectly responsible for most human epidemics [13]. With economic and transportation development, human activities are increasingly and persistently disturbing their habitats, potentially resulting in contact and communication between different populations of *M. himalayana* and facilitating the transmission of *Y. pestis*, thereby increasing the risk of plague outbreaks [59]. In the DFR gene typing of 245 *Y*. *pestis* strains in the Sanjiangyuan region, the predominant genotypes identified were type 5 and type 8. Type 5 strains were found in the YS, GL, and HU groups, whereas type 8 strains were distributed in the YS, GL, and HA groups. However, types 10, 14, 30, and 36 were found exclusively in the YS group [11]. According to SNP typing results, 78 strains of *Y. pestis* were distributed among three SNP branches: 1.IN, 2.ANT and 0.PE. The 1.IN branch was the dominant population in the natural plague foci of the Sanjiangyuan region, comprising all *Y. pestis* strains associated with *M. himalayana*. The 2.ANT and 0.PE branches were scattered across Chengduo, Nangqian, and Qumalai counties in the YS group [37].

This study demonstrates that the GL group exhibits higher abundance in pathways related to viral diseases, digestive function, and immune system processes, with a notable enrichment of highly aggregated drug-resistant genes. The functional gene annotations show significant differences compared to the other three groups, indicating that the *M. himalayana* population in the GL group possesses enhanced immunity capabilities. Conversely, no unique genotypes of *Y. pestis* were identified in the Guoluo region. Historical plague surveillance data reveal that marmot populations in Dari County, Banma County, Jiuzhi County, and Gande County have never experienced animal plague outbreaks. A speculative hypothesis proposes that the functional composition of the gut microbiota of *M. himalayana* and genotypes of *Y. pestis* may influence the potential for plague outbreaks. However, further experimental and longitudinal studies are required to validate whether and how the gut microbiota functions in *M. himalayana* influences plague dynamics.

## 5 Conclusion

The gut microbiota of *M. himalayana* is predominantly composed of *Firmicutes* and *Bacteroidota*, with *Alistipes*, *Bacteroides*, and *Clostridium* as key genera. A structured and metabolically active microbial community facilitates the physiological adaptation of *M. himalayana* to the extreme conditions of the Qinghai-Xizang Plateau. The primary antibiotic resistance genes present in high abundance within the gut microbiota of *M. himalayana* include *MCR-1.2*, *SHV-100*, *MexH*, and *vanC*, all of which are genes associated with bacterial antibiotic resistance. As a critical component of global microbial biodiversity, research on *M. himalayana’s* gut microbiota not only contributes to regional biodiversity preservation but also enhances our understanding of ecosystem stability.

## Ethics statement

This study was reviewed and approved by the Ethics Committee of the Qinghai Institute for Endemic Disease Prevention and Control (approval number QDB 2019–0009). Informed consent was obtained from all participants.

## Acknowledgements

This work was supported by the Key 10.13039/100006190Research and Development and Transformation Plan of Qinghai Province, China (Grant No. 2025-QY-208); 10.13039/501100001809National Natural Science Foundation of China, China (Grant No. 82460660); Entrepreneurship and Innovation Team for Disease Vector Prevention and Control Research in Qinghai, China.

## Conflict of interest statement

The authors declare that there are no conflicts of interest.

## Author Contributions

**Ying Ma:** Writing – original draft, Investigation, Conceptualization. **Ziyan Li:** Writing – original draft, Data curation. **Pengbo Liu:** Visualization, Formal analysis. **Youwen Wei:** Investigation, Data curation. **Ke Jiang:** Data curation. **Yujuan Yue:** Visualization. **Aiping Zhang:** Funding acquisition. **Wenlong Wang:** Investigation. **Lingwen Li:** Investigation. **Penghui Zhang:** Investigation. **Xingyue Gu:** Investigation. **Qiyong Liu:** Writing – review & editing, Supervision, Conceptualization. **Liang Lu:** Writing – review & editing, Supervision, Visualization, Formal analysis, Conceptualization.
