# Supplementary material for: Metagenomics research on the gut microbiota of the Marmota himalayana of the Sanjiangyuan National Nature Reserve in Qinghai Province, China
Source: Biosaf Health. 2025 Sep 8;7(5):281–94. doi: 10.1016/j.bsheal.2025.09.003 (PMC12624539; doi:10.1016/j.bsheal.2025.09.003)
Supplement: Supplementary Data 1 [file mmc1.docx]

**Fig. S1.**

Petal plot of gene number of intestinal microflora among groups of *M. himalayana* in Sanjiangyuan National Nature Reserve.

Abbreviations: *M. himalayana*, *Marmota himalayana*; GL, Guoluo Prefecture; YS, Yushu prefecture; HA, Hainan Prefecture; HU, Huangnan Prefecture.

**Fig. S2.**

Petal plot of gene number of intestinal microflora among groups of *M. himalayana* in Sanjiangyuan National Nature Reserve.

Abbreviations: *M. himalayana*, *Marmota himalayana*.

**Fig. S3.**

Stacked histograms of the relative abundance of intestinal microflora at phylumgenus level (A) and genus level (B) of *M. himalayana* in Sanjiangyuan National Nature Reserve*.*

Abbreviations: *M. himalayana*, *Marmota himalayana*; GL, Guoluo Prefecture; YS, Yushu prefecture; HA, Hainan Prefecture; HU, Huangnan Prefecture

**Fig. S4.**

Boxplot of ARO abundance difference among intestinal microflora of different groups of *M. himalayana* in Sanjiangyuan National Nature Reserve.

Abbreviations: ARO, Antibiotic Resistance Ontology; *M. himalayana*, *Marmota himalayana*; GL, Guoluo Prefecture; YS, Yushu Prefecture; HA, Hainan Prefecture; HU, Huangnan Prefecture.
